# Immunomodulatory Effects of Hydrolyzed Seawater Pearl Tablet (HSPT) on Th1/Th2 Functionality in a Mice Model of Chronic Obstructive Pulmonary Disease (COPD) Induced by Cigarette Smoke

**DOI:** 10.1155/2020/5931652

**Published:** 2020-11-18

**Authors:** Zhenxing Chen, Qiangqiang Yan, Zhongmin Zhang, Taijin Lan, Peng Liu, Siyin Han, Yong Lin, Jiang Lin

**Affiliations:** ^1^College of Basic Medicine, Guangxi University of Chinese Medicine, Nanning 530200, China; ^2^College of Pharmacy, Guangxi University of Chinese Medicine, Nanning 530200, China; ^3^Independent Researcher, Beihai 536000, China

## Abstract

Chronic obstructive pulmonary disease (COPD) is predicted to become the third leading cause of death around the world. The present study is designed to investigate whether hydrolyzed seawater pearl tablet (HSPT) has immunoregulatory effects on the Th1/Th2 functionality in cigarette smoke-induced COPD model mice. The determination of the amino acid composition of HSPT was carried out by high-performance liquid chromatography (HPLC) with precolumn phenylisothiocyanate (PITC) derivatization. COPD model mice were constructed by cigarette smoking (CS) treatment and HSPT was administered. HSPT inhibited the infiltration of inflammation in the airway of the lung, reduced influx of eosinophils (EOSs), lymphocytes (LYMs), neutrophils (NEUs), and macrophages (MACs) in the bronchoalveolar lavage fluid (BALF), decreased the levels of IFN-*γ*, IL-2, IL-4, and IL-10 in the serum and lung, and decreased the expression of aforementioned cytokines in the spleen and lung in CS-treated mice. Besides, HSPT also had the ability to reduce the amount of CD3^+^CD4^+^ T cells and modulate the Th1/Th2 balance. Taken together, this study supports the consensus that CS is a critical factor to induce and aggravate COPD. HSPT could regulate the balance of Th1/Th2 in CS-induced COPD model mice, indicating its effects on inhibiting the development of COPD.

## 1. Introduction

Chronic obstructive pulmonary disease (COPD) is an incurable lung disease characterized by progressive airflow obstruction involving emphysematous destruction of lung parenchyma and mucus hypersecretion with chronic bronchitis [1, 2]. COPD has been recognized as a major public health problem and might become a considerable burden worldwide in the near future [3]. It is a public burden in global health and will become the third leading cause of death in the world by 2030 [4]. The most common symptoms of COPD are dyspnea, cough, and sputum production, and less common but troublesome symptoms are wheezing, chest tightness, and chest congestion [5]. Apart from physical impairment, patients with COPD carry a substantial mental burden related to their disease and its symptoms [6]. COPD is characterized by persistent and progressive airway inflammation [7]. Cigarette smoking (CS) is a leading cause of COPD [8], with other factors including ambient air pollution, airway hyperresponsiveness, and allergy [9]. The Global Initiative for Chronic Obstructive Lung Disease (GOLD) recommends long-acting bronchodilators for the management of patients with stable COPD [10]; however, the currently available drugs only relieve the symptoms of COPD. In addition, existing medical treatments for the management of very severe COPD still have limitations [11]. Treatment of COPD over the past decades of management includes pharmacological and nonpharmacological interventions with the target to improve functional performance and quality of life, control symptoms, and exacerbations, whereas clinical practice causes unsatisfactory results as compared with the expectations [12]. Therefore, the development of novel strategies and new pharmacologic agents to treat COPD is necessary.

CD4^+^ T helper lymphocytes (Th cells) play a critical role in the dysfunction of the immune, which contributes to the progress of COPD [13]. When the body encounters external stimulation, Th cells are activated to develop into a subset of effector cells, involving Th1 and Th2 cells [14]. The Th1 and Th2 cells play critical roles in the regulation of cellular and humoral immune responses. The disorder of Th1/Th2 cell differentiation makes the immune response imbalanced in the body, leading to persistent inflammatory reactions, airway remodeling, and emphysema [15]. The Th1 subset secretes proinflammatory cytokines, such as IFN-*γ* and IL-2, and the Th2 subset secretes IL-4 and IL-10 [16]. When COPD appeared, the Th1/Th2 balance is broken, showing the imbalance of inflammatory cytokines secretion [17].

Pearl has been traditionally and popularly used as both functional food and medicine for thousands of years. In 2002, the Ministry of Health in China issued the “Further Regulating Health Care Product Raw Materials Management Notice,” including pearl on the “List of Items Used in Health Care Products.” It has been applied to cure insomnia, palpitations, convulsions, epilepsy, and ulcers for thousands of years. According to the *Pharmacopoeia of the People's Republic of China*, the pearl is formed in *Pteria martensii* Dunker (Pteriidae), *Hyriopsis cumingii* Lea, *Cristaria plicata* Leach (Unionidae), etc. Modern research has proved that the clinical application of pearl is due to its antioxidant, antiaging, antiradiative, and tonic activities and wound healing effect [18–20]. Pearl is mainly composed of calcium carbonate and magnesium carbonate, which accounted for 95% of the total weight [21]. Other inorganic molecules such as silica, calcium phosphate, aluminum oxide, and ferric oxide, as well as some trace elements such as sodium, selenium, potassium, magnesium, manganese, and boron, can also be found in pearl powder [22]. It also contains 15–18 essential amino acids including aspartic (Asp), glutamic (Glu), lysine (Lys), valine (Val), methionine (Met), threonine (Thr), phenylalanine (Phe), leucine (Leu), tryptophan (Trp), and histidine (His) [23]. The major nutrients in pearl powder are enriched with proteins, peptides, and amino acids, making great contributions to the body's bioactivity including improving the antioxidant oxidation resistance defense system [24].

Hydrolyzed seawater pearl tablet (HSPT) is derived from pearl which was hydrolyzed by enzymatic proteases following our patented technology (Jiang Lin, et al. 2000, Chinese patent, ZL95106283.2). To date, aiming at explaining the clinical practices, finding the bioactive property, and providing insight into the mode of action, our research group have conducted a systematic study on the extracts and active component form pearl [25–29]. In a previous study, we had found that HSPT could improve the body's immunity by regulating the numbers of T cells [25]. The finding provided a basis for the clinical application of HSPT for respiratory diseases; additionally, it indicated the important role of HSPT in the treatment of COPD.

However, to our knowledge, it is still unknown whether HSPT reduces inflammation in the lungs of COPD by correcting the Th1/Th2 balance. This study was designed to evaluate the effect of hydrolyzed seawater pearl powder on COPD in mice. It was hypothesized that the rebalance of Th1/Th2 cells might represent essential regulatory mechanisms potential in the effects of HSPT.

## 2. Materials and Methods

### 2.1. Materials and Reagents

Hydrolyzed seawater pearl tablets (production approval number: 450522020014, product number: 20180102) were kindly donated by Baozhulin Ocean Technology Co., Ltd. (Beihai, China) and used throughout the study. Amino acid standards (purity 99%) were purchased from Sepax Technologies, Inc. (Suzhou, China). The internal standard (IS) 2-aminohexanoic acid was purchased from EKEAR Biological Technology Co., Ltd. (Shanghai, China) with a purity of 99%. N-Acetylcysteine was purchased from Zambon Pharmaceutical Company (Hainan, China). All other reagents and chemicals used in the study were of analytical grade or better.

### 2.2. Amino Acid Analysis

Sample preparation was performed with slight improvement according to the previously described method [2]. The HSPT powder of 100 g was immersed with 10 times of water and stirred and allowed to react at 4°C for 12 h. Then, the solution was filtered, and the filtrate was freeze-dried to obtain the HSPT protein sample. The amino acid analysis of the sample was analyzed using a 1260 Infinity HPLC-ELSD (Agilent Technologies, Santa Clara, CA, USA) equipped with a Sepax AAA column (4.6 × 150 mm, 5 *μ*m; Sepax Technologies, Inc., Suzhou, China). The column temperature was maintained at 36°C. The mobile phase consisted of 100 mM sodium acetate in 7% acetonitrile at pH 6.5 (A) and 80% acetonitrile in water (B) with a gradient program as follows: 0 ∼ 15 min, 100% ∼ 85% A; 15 ∼ 18 min, 85% ∼ 76% A; 18 ∼ 25 min, 76% ∼ 60% *A*; 25 ∼ 30 min, 60% *A*; 30.01 ∼ 40 min, 0% *A*. Standard or sample solution (5 *μ*L) was injected into the HPLC system, and the flow rate was kept at 1.0 mL/min for 70 min. The detection wavelength was 254 nm. Chromatographic analysis of standard and sample solution was performed in triplicate, and data were recorded and processed with OpenLab CDS 2.2 software.

### 2.3. Animals and Ethics Statement

Female 6- to 8-week-old C57BL/6 mice were purchased from Hunan Slack Jingda Experimental Animal Company Ltd. (license no. SCXK (Xiang) 2019-0004). All experimental procedures were performed in accordance with the National Institutes of Health (NIH) Guide for the Care and Use of Laboratory Animals. The study protocol was approved by the animal ethics committee of the Guangxi University of Chinese Medicine. All animal experiments were carried out in Guangxi University of Chinese Medicine Laboratory Animal Research Center (license no. SYXK (Gui) 2019-0001). Mice were housed five per cage and maintained at constant temperature (22 ± 2°C) and constant humidity (55 ± 5%) with a 12 h light/dark cycle. The animals were fed with forage and clean water *ad libitum*. All experimental procedures followed the National Animal Welfare Law of China. Prior to the study experiments, animals were provided adaptive feeding for 7 days.

### 2.4. Grouping and Administration

A total of 60 mice were randomly divided into six groups (*n* = 10 per group): Control group (Con group), which received normal saline and was not exposed to CS; cigarette smoke-exposed group (CS group), which received normal saline and was exposed to CS; and cigarette smoke-exposed N-acetylcysteine (NAC, positive drug) group (NAC group), which received 150 mg/kg NAC (q.o.d) and was subsequently exposed to CS. Based on clinical usage (2.5 g/day for adults) and the Meeh-Rubner equation of dose conversion, the human equivalent dosage of HSPT was about 370 mg/kg/day for mice. Therefore, cigarette smoke-exposed medium-dose HSPT group (HSPT (M) group) received 370 mg/kg HSPT (q.o.d). In addition, the low dose and high dose of HSPT for mice were 185 and 740 mg/kg (q.o.d), which were 0.5 and 2 times those of humans corresponding to HSPT. HSPT was resuspended with distilled water and administrated by gavage with a volume of 10 mL/kg. After the administration of HSPT, all mice were subsequently exposed to CS.

### 2.5. Induction of COPD in Mice

**Figure 1:**
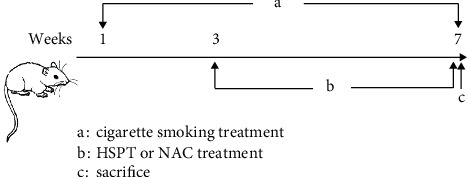
Cigarette smoke exposure protocol with HSPT or NAC intervention.

Induction of COPD in mice was performed according to the previously described method with minor modification [30]. Cigarette smoke exposure was done for seven consecutive weeks, six days per week. In the 3rd week, HSPT or NAC was administered orally to mice until the end of the experiment (Figure 1). Briefly, female C57BL/6 mice were grouped as in the methods mentioned above, and all the intervention group mice received the drug by oral administration at the abovementioned doses 1 h before CS exposure and then exposed to the smoke of five commercially available filtered cigarettes (Marlboro, a cigarette containing 10.0 mg tar and 0.8 mg nicotine, Philip Morris) for 30 min twice daily, 6 days per week for 7 weeks. All animals fasted for 12 h and were free to drink water prior to the last CS exposure. At last, all animals were sacrificed to collect bronchoalveolar lavage fluid (BALF), lung and spleen tissues, and whole blood samples.

### 2.6. Safety Evaluation of HSPT

The body weight of mice was recorded every three days during the experiment. In addition, the spleen and lung of treated mice were collected and weighed after the last CS treated. The visceral indexes (spleen and lung) were investigated for the safety evaluation of HSPT. The visceral indexes were calculated using the following formula: visceral indexes (%) = (viscera weight/body weight) × 1000‰.

### 2.7. Analysis of Bronchoalveolar Lavage Fluid (BALF) Samples

Tracheas were cannulated after the collection of whole blood in mice. BALF was collected with 4 repeated washes of excised lungs using 2 ml saline in total. Then, the BALF was centrifuged at 3000 rpm for 10 min at 4°C. After centrifugation, the precipitates were collected and resuspended with 100 *μ*L normal saline, and 20 *μ*L was used for cell counting. The remaining cells were resuspended with another 1 mL normal saline solution to make cell smear and further stained with Diff-Quik staining solution (Solarbio, Beijing, China). Cells were visualized under the microscope, and the number of staining cells was determined by counting at least 200 cells. Differential cell types were classified and counted according to their cellular morphology.

### 2.8. Histopathology Analysis

After BALF was collected, the upper left lung bole was removed and then immediately fixed in 4% paraformaldehyde for 24 h. Subsequently, the tissues were washed with running water and immersed in a test tube with 30 ml of deionized water for 6 h. Then, tissues were fixed in 70% ethanol overnight and dehydrated through an ethanol series up to 100% ethanol. And then specimens were embedded in paraffin. 5 *μ*m sections of fixed embedded tissues were cut and stained with Hematoxylin and Eosin staining (H&E) for histopathological examination.

### 2.9. ELISA Assay

Mice IFN-*γ* ELISA kit, IL-2 ELISA kit, IL-4 ELISA kit, and IL-10 ELISA kit (all from Solarbio, Beijing, China) were used to measure the levels of IFN-*γ*, IL-2, IL-4, and IL-10 in serum according to the manufacturer's instructions. The standard curve was made with IFN-*γ*, IL-2, IL-4, and IL-10, respectively. The optical densities were measured by the absorbance at 450 nm, and results were expressed as pg/ml.

### 2.10. Western Bolt

Lung tissues were minced and homogenized in ice-cold RIPA lysis buffer, followed by centrifugation in a cooling centrifuge (12,000 rpm for 45 min at 0–4°C) to remove the debris, and the supernatant was used for Western blot analysis. Based on the concentration measured by BCA assay, an equivalent amount of each protein sample was subjected to a 15% SDS-PAGE gel electrophoresis and transferred to nitrocellulose membranes. Membranes were blocked with 5% skim milk powder for 1 h; sequentially, primary antibodies (all from Biosynthesis Biotechnology Co., Ltd., Beijing, China) were incubated at room temperature for 3 h. After incubation, the membranes were subsequently incubated for 1 h at room temperature with the secondary antibodies conjugated with horseradish peroxidase. The protein bands were visualized following an enhanced chemiluminescence method using an ELC kit.

### 2.11. RNA Preparation and qRT-PCR

Mice total RNA of lung and spleen tissues were extracted using Trizol Reagent (Invitrogen) to prepare cDNA. Reverse transcription-PCR (RT-PCR) was performed using a two-step method. Then, PCR amplification was carried out using Fast Start Universal SYBR Green Master (ROX; Roche, Basel, Switzerland) and relevant primers. The primers were synthesized by Huada Gene Company (Beijing, China). The primer sequences validated in this study are summarized in Table 1.

The StepOnePlus real-time PCR system (ABI, Carlsbad, CA) was used to detect the real-time change in cDNA. The thermocycling conditions were as follows: 1 cycle of 95°C for 30 seconds and 40 cycles of 95°C for 3 seconds and 60°C for 30 seconds. Cycle threshold (Ct) values were obtained graphically for both different target genes and *β*-actin. 2^−ΔΔCt^ values were calculated to represent the amounts of different target genes.

### 2.12. Flow Cytometric Analysis

Single-cell suspensions were prepared from spleen tissues of mice. In brief, spleen tissues were dispersed by repetitive suction and passed through a 70 *μ*m cell strainer. The lymphocytes from the spleen were isolated and purified using a lymphocyte separation solution (Solarbio, Beijing, China) as previously described [31]. After that, splenocytes were washed with PBS and resuspended in complete medium at a density of 1 × 10^7^ cells/mL for intracellular staining.

A mice Th1/Th2 staining kit (Multi Science (LIANKE), Hangzhou, China) was used for flow cytometric analysis. In the mice Th1/Th2 staining kit, FITC-anti-Mouse CD3*ε* and PerCP-Cy5.5-anti-Mouse CD4 were used to identify CD3^+^ or CD4^+^ T cell types. We captured 30,000 events of each sample, and dead cells were gated out depending on FSC and SSC. First, CD3^+^ or CD4^+^ T cells were gated based on the negative blank (only cells) and the positive dye (FITC-anti-Mouse CD3*ε*). Subsequently, depending on the negative blank (only cells) and the positive dye, we divided the panel into four quadrants (Q1-4). Q2 quadrants represent CD3^+^CD4^+^ T cells. In addition, PE-anti-Mouse IFN-*γ* and APC-anti-Mouse IL-4 were used to detect the intracellular staining of CD3^+^CD4^+^ IFN-*γ*^+^ and CD3^+^CD4^+^ IL-4^+^ T cell types. According to the instruction, 250 *μ*L splenocyte suspension at a density of 1 × 10^7^ cells/mL was stimulated with a leukocyte activation cocktail (PMA/ionomycin mixture and BFA/monensin mixture) for 5 h; then 100 *μ*L of splenocyte suspension was transferred into a flow cytometry tube. Splenocytes were fixed/permeabilized using the FIX & PERM kit, followed by incubation with FITC-anti-Mouse CD3*ε*, PerCP-Cy5.5-anti-Mouse CD4, PE-anti-Mouse IFN-*γ*, and APC-anti-Mouse IL-4 to identify CD3^+^ CD4^+^ IFN-*γ*^+^ and CD3^+^CD4^+^ IL-4^+^ T cell types, respectively.

The analysis of cell markers was all performed using a BD FACS Calibur® flow cytometry system (BD Bioscience, San Jose, CA, USA). Flow cytometry data analysis was finally done and displayed with FlowJo (FlowJo, LLC, USA) and OriginPro 8.5 (OriginLab, MA, USA).

### 2.13. Statistical Analysis

Data were expressed as the mean ± SEM. Each experiment was repeated at least three times. Statistical analysis was performed with SPSS 22.0 software (SPSS, Chicago, USA) by using one-way ANOVA and least significant difference (LSD) tests. *P* < 0.05 was considered statistically significant.

## 3. Results

### 3.1. Amino Acid Analysis

**Figure 2:**
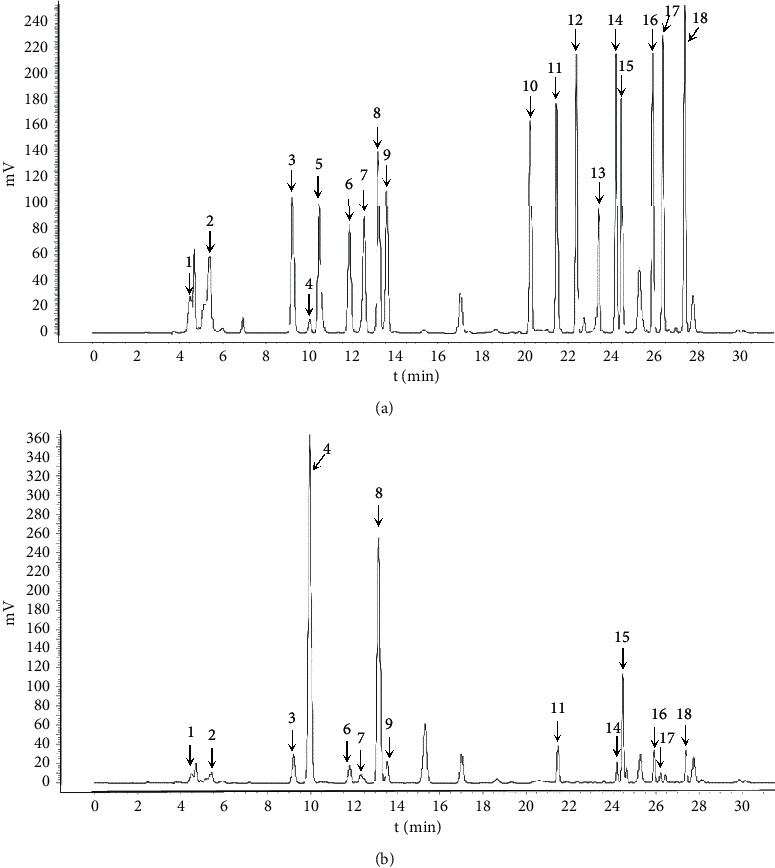
Amino acid analysis. Amino acid standards (a). HSPT protein (b). 1: asparagine (Asp), 2: glutamic acid (Glu), 3: serine (Ser), 4: glycine (Gly), 5: histidine (His), 6: arginine (Arg), 7: threonine (Thr), 8: alanine (Ala), 9: proline (Pro), 10. tyrosine (Tyr), 11. valine (Val), 12: methionine (Met), 13: cysteine (Cys), 14: isoleucine (Ile), 15: leucine (Leu), 16: phenylalanine (Phe), 17: tryptophan (Try), 18: lysine (Lys).

The yield for the obtained proteins for HSPT is 0.23%. The amino acid composition was determined by HPLC combined with precolumn PITC derivatization and the chromatogram is shown in Figure 2. It was clear that the HSPT protein sample contained several amino acids, mainly including asparagine (Asp), glutamic acid (Glu), serine (Ser), glycine (Gly), arginine (Arg), threonine (Thr), alanine (Ala), proline (Pro), valine (Val), isoleucine (Ile), leucine (Leu), phenylalanine (Phe), tryptophan (Try), and lysine (Lys).

### 3.2. HSPT without Any Toxicity Reactions

**Figure 3:**
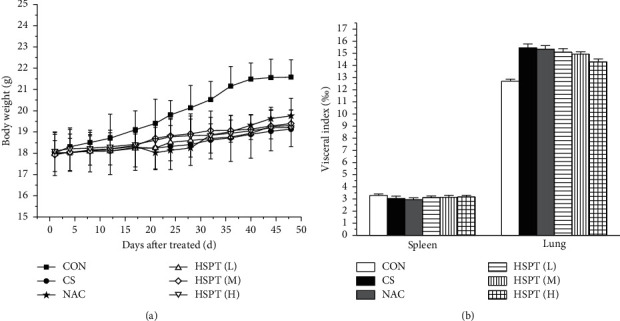
Safety evaluation of HSPT. The changes in body weight for each group (a). Visceral indexes (spleen and lung) for each group (b). Data are expressed as means ± SEM n = 10 in each group.

Body weight changes and visceral indexes were selected to evaluate the safety of HSPT. During the experiment, the average weight of mice increased slightly. Compared with the Con group, the CS group showed a significant loss of body weight. However, the mouse body weights in the intervention groups were increased to a certain extent (Figure 3(a)). With the visceral indexes results, we found that no marked difference in spleen index was observed in each group, whereas a marked increase in lung index was observed in the CS group and HSPT could decrease the level of lung index (Figure 3(b)), which implied that HSPT could ameliorate the CS-induced lung swelling. These results, taken together, illustrate that HSPT is safe to use in COPD treatment.

### 3.3. HSPT Inhibited Infiltration of Inflammatory Cells into the Lung

**Figure 4:**
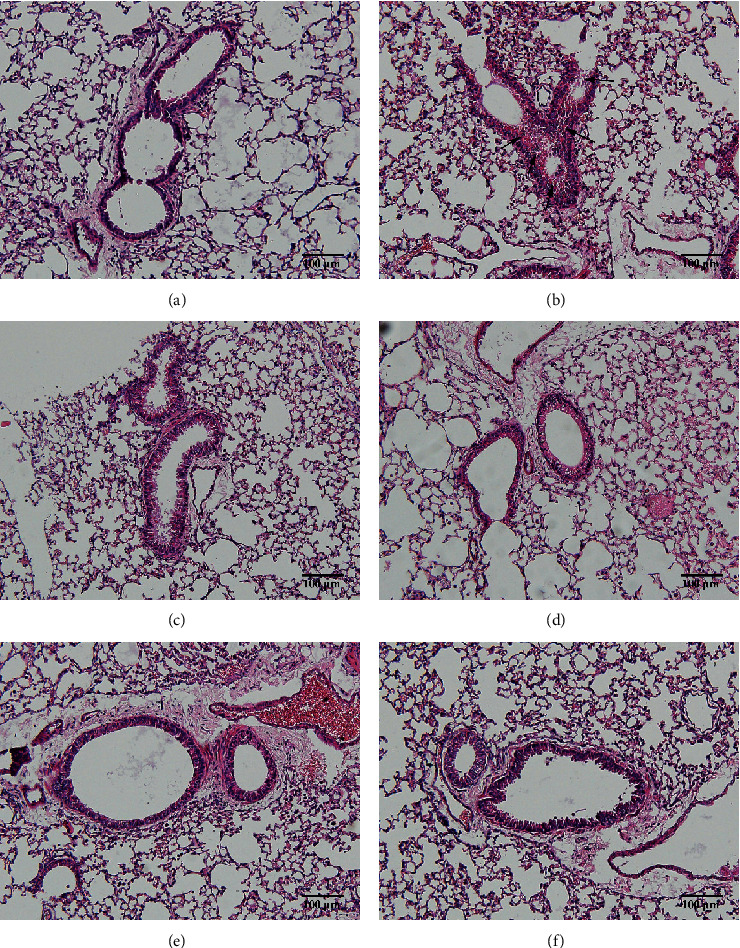
HSPT inhibited airway inflammation into the lung. Administration of HSPT or NAC markedly decreased the airway inflammation into lung: the lungs were removed 1 h after the last CS treatment. Sections were stained by H&E (200x). Con group (a), CS group (b), NAC group (c), HSPT (L) group (d), HSPT (M) group (e), and HSPT (H) group (f). The experiment was repeated six times; a representative result was shown. *n* = 10 in each group. Arrows indicate the inflammatory in the airway.

Compared with the control group, the degree of inflammatory cell infiltration in the airway mucosa that was treated with CS was increased, basement membrane and smooth muscle became thicker, and the tracheal lumen also had stenosis at different levels. Mice treated with NAC showed a marked reduction in the inflammatory cell infiltration in the lung. It is important to note that HSPT inhibited the infiltration in the airway mucosa in a dose-dependent manner (Figure 4).

### 3.4. HSPT Reduced Influx of EOSs, LYMs, NEUs, and MACs in the BALF

**Figure 5:**
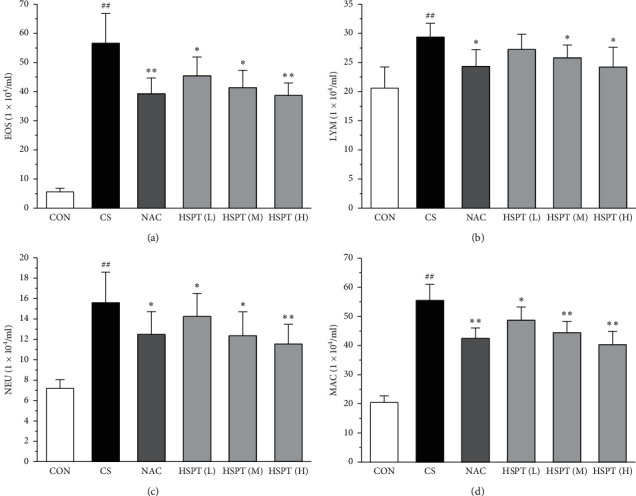
HSPT reduced the influx of EOSs, LYMs, NEUs, and MACs in the BALF. Administration of HSPT or NAC markedly decreased the amount of migrated inflammatory cells: EOSs (a), LYMs (b), NEUs (c), and MACs (d) in the BALF from the CS mice. The BALF cells were collected 1 h after the last CS treatment. The different cell types were enumerated. Data are expressed as means ± SEM (*n* = 10), ^##^*P* < 0.01 vs the Con group, ^*∗*^*P* < 0.05, ^*∗∗*^*P* < 0.01 vs the CS control.

To determine the effects of HSPT on CS-induced pulmonary inflammation responses in mice, the inflammatory cell counts in BALF were assayed. In the CS group, mice showed an increased level of inflammatory cells (EOSs, LYMs, NEUs, and MACs) in BALF when compared with levels in control group mice. However, the administration of NAC or HSPT obviously decreased the levels of inflammatory cells (EOSs, LYMs, NEUs, and MACs) in BALF from the CS mice (Figures 5(a)–5(d)).

### 3.5. HSPT Decreased IFN-*γ*, IL-2, IL-4, and IL-10 in the Serum

**Figure 6:**
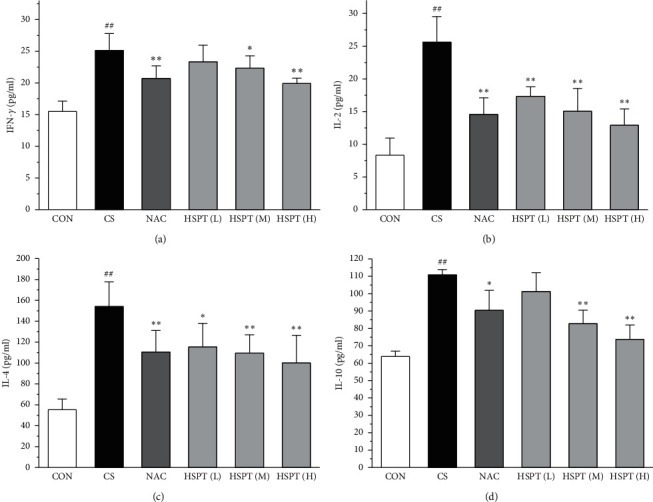
HSPT affects the levels of cytokines in the serum. Administration of HSPT or NAC markedly decreased the amounts of inflammatory cytokines: IFN-*γ* (a), IL-2 (b), IL-4 (c), and IL-10 (d) in the serum from the CS mice. The serum was collected 1 h after the last CS treatment. The different cell types were enumerated. Data are expressed as means ± SEM (*n* = 10), ^##^*P* < 0.01 vs the Con group, ^*∗*^*P* < 0.05, ^*∗∗*^*P* < 0.01 vs the CS control.

The levels of IFN-*γ* and IL-2, the principal Th1 cytokines, were increased in CS mice when compared to the Con mice. Moreover, CS mice developed a marked elevation of Th2 cytokines (IL-4 and IL-10) as compared with the control mice. These results indicated the imbalance of Th1/Th2 in CS mice. However, HSPT or NAC treatment significantly decreased the Th1 cytokines (IFN-*γ* and IL-2) and Th2 cytokines (IL-4 and IL-10) in CS mice. These pieces of evidence indicated that HSPT regulated the Th1/Th2 balance (Figures 6(a)–6(d)).

### 3.6. HSPT Altered the Protein Expression Levels of IFN-*γ*, IL-2, IL-4, and IL-10 in the Lung

**Figure 7:**
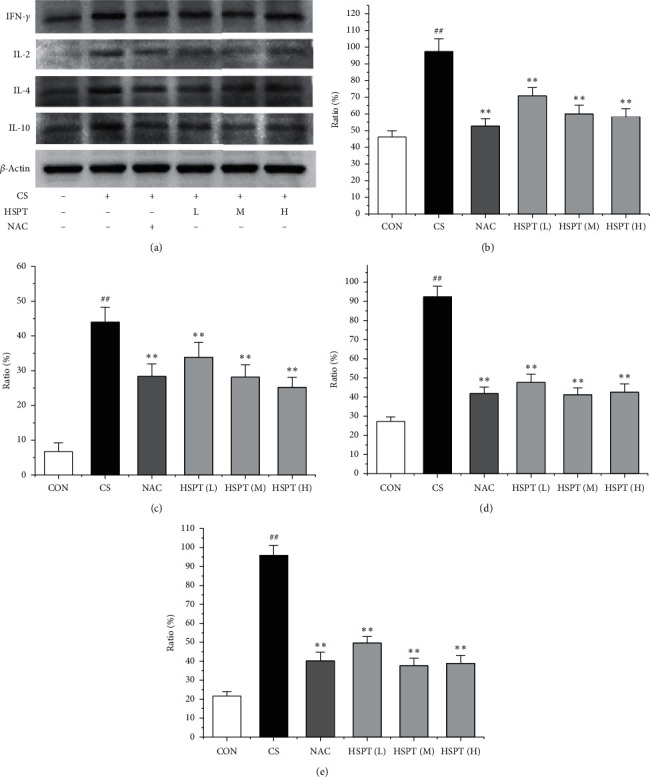
HSPT altered the protein expression levels of cytokines in the lung. Administration of HSPT or NAC markedly decreased the amounts of inflammatory cytokines: protein bands of all detective cytokines (a); IFN-*γ* (b), IL-2 (c), IL-4 (d), and IL-10 (e) in the lung from the CS mice. The lung tissues were collected 1 h after the last CS treatment. The different cell types were enumerated. Data are expressed as means ± SEM (*n* = 10), ^##^*P* < 0.01 vs the Con group, ^*∗*^*P* < 0.05, ^*∗∗*^*P* < 0.01 vs the CS control.

The protein expression levels of IFN-*γ*, IL-2, IL-4, and IL-10 in the lung were detected with Western bolt (Figure 7(a)). The detective inflammatory cytokines in CS mice showed a significant increase compared with the mice in the Con group. However, administration with HSPT obviously decreased the protein expression level of inflammatory cytokines dose-dependently, when compared to the mice in the CS group (Figures 7(b)–7(e)).

### 3.7. HSPT Altered the mRNA Expression Levels of IFN-*γ*, IL-2, IL-4, and IL-10 in the Lung and Spleen

**Figure 8:**
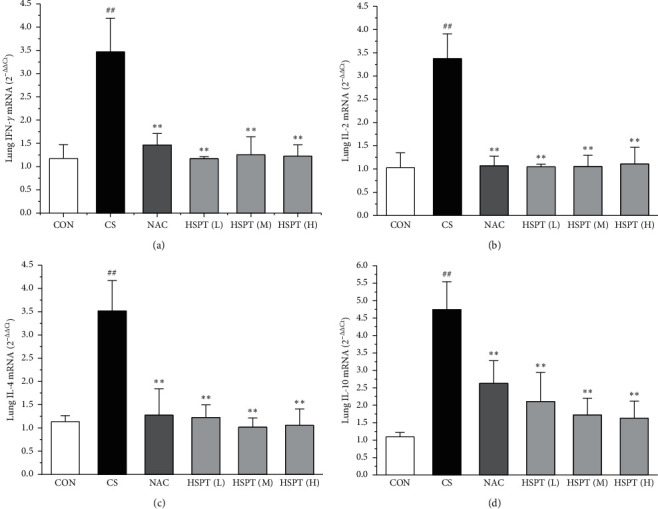
HSPT altered the mRNA expression levels of cytokines in the lung. Administration of HSPT or NAC markedly decreased the amounts of inflammatory cytokines: IFN-*γ* (a), IL-2 (b), IL-4 (c), and IL-10 (d) in the lung from the CS mice. The lung tissues were collected 1 h after the last CS treatment. The different cell types were enumerated. Data are expressed as means± SEM (*n* = 10), ^##^*P* < 0.01 vs the Con group, ^*∗*^*P* < 0.05, ^*∗∗*^*P* < 0.01 vs the CS control.

**Figure 9:**
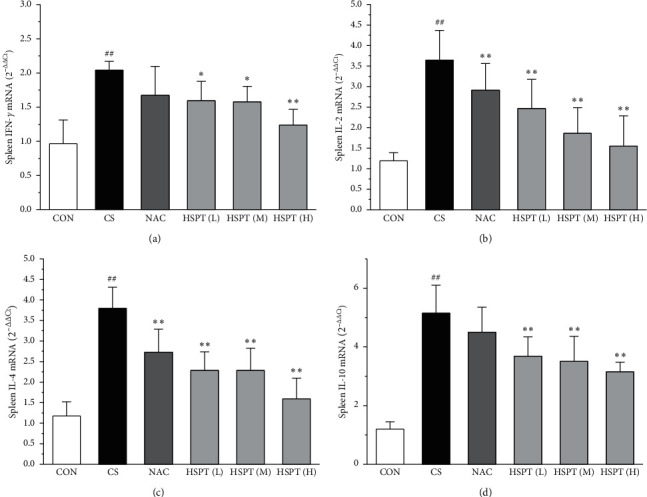
HSPT altered the mRNA expression levels of cytokines in the spleen. Administration of HSPT or NAC markedly decreased the amounts of inflammatory cytokines: IFN-*γ* (a), IL-2 (b), IL-4 (c), and IL-10 (d) in the spleen from the CS mice. The spleen tissues were collected 1 h after the last CS treatment. The different cell types were enumerated. Data are expressed as means ± SEM (*n* = 10), ^##^*P* < 0.01 vs the Con group, ^*∗*^*P* < 0.05, ^*∗∗*^*P* < 0.01 vs the CS control.

The mRNA expression levels of inflammatory cytokines for Th1 and Th2 in the lung and spleen were detected with quantitative RT-PCR (Figures 8 and 9). We found that the expression patterns of the aforementioned genes (IFN-*γ*, IL-2, IL-4, and IL-10) in the spleen of each group were similar to those in the lung. In addition, it showed similar results as with cytokine levels in serum and protein expression in the lung of IFN-*γ*, IL-2, IL-4, and IL-10. The detective inflammatory cytokines in CS mice showed a significant increase compared with the mice in the Con group. However, administration with HSPT obviously decreased the mRNA expression level of inflammatory cytokines dose-dependently, when compared to the mice in the CS group.

### 3.8. HSPT Influenced T Lymphocyte Subpopulation in the Spleen

**Figure 10:**
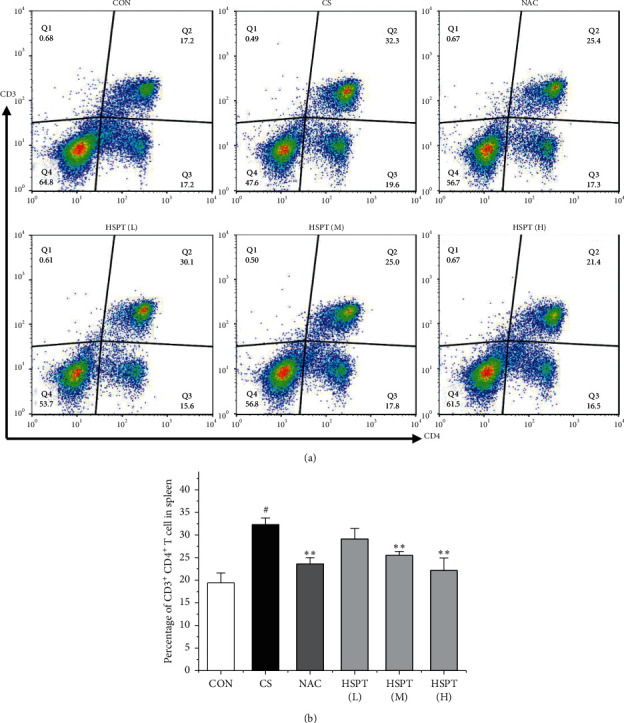
HSPT influenced T lymphocyte subpopulation in the spleen. Administration of HSPT or NAC influenced the T lymphocyte subpopulation in the spleen: the spleen tissues were collected 1 h after the last CS treatment. Single-cell suspensions of spleen were prepared as described in the materials and methods, stained with anti-CD3 and anti-CD4 antibodies, and then measured by flow cytometry. Quantitative analysis of CD3^+^CD4^+^ T cells (a) was shown, and the percentage of CD3^+^CD4^+^ T cells (b) was calculated. Data are expressed as means ± SEM (*n* = 10), ^#^*P* < 0.05, ^##^*P* < 0.01 vs the Con group, ^*∗*^*P* < 0.05, ^*∗∗*^*P* < 0.01 vs the CS control.

Inflammation is thought to play a major role in the pathogenesis of COPD [10]. T lymphocytes and T lymphocyte subpopulation play an important and diverse role in the establishment and suppression of inflammation [32]. We investigated the proportion of CD3^+^CD4^+^ T lymphocytes in splenic total lymphocyte. In our study, it was revealed that CS caused a significant increase of CD3^+^CD4^+^ T lymphocytes ratio in spleen tissues to fight inflammation (Figure 10). What's more, we found that HSPT treatment decreased the splenic CD3^+^CD4^+^ T lymphocytes ratio in a dose-dependent manner (Figure 10(b)).

### 3.9. HSPT Influenced T Lymphocyte Subpopulations and Impairs the Th1/Th2 Balance In Vivo

**Figure 11:**
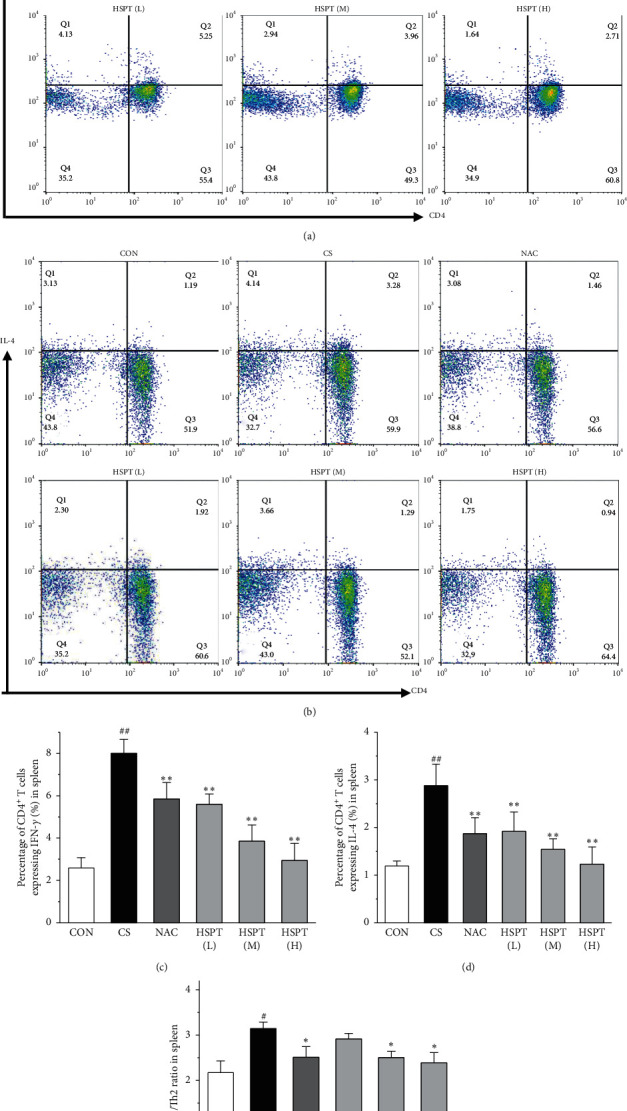
Percentages of IFN-*γ*^+^ T cells and IL-4^+^ T cells in splenocytes. Administration of HSPT or NAC influenced the T lymphocyte subpopulation in the spleen: the spleen tissues were collected 1 h after the last CS treatment. Single-cell suspensions of spleen were prepared as described in the materials and methods, stained with FITC-anti-Mouse CD3*ε*, PerCP-Cy5.5-anti-Mouse CD4, PE-anti-Mouse IFN-*γ*, and APC-anti-Mouse IL-4 antibodies and then measured by flow cytometry. The left quadrants represent CD3^+^CD4^−^ and the right quadrants represent CD3^+^CD4^+^ T cells ((a), (b)). Percentages of CD3^+^CD4^+^ IFN-*γ*^+^ Th1 cells (c) and CD3^+^CD4^+^ IL4^+^ Th2 cells (d) were quantitative analyzed and Th1/Th2 ratio was calculated (e). Data are expressed as means ± SEM (*n* = 10), ^#^*P* < 0.05, ^##^*P* < 0.01 vs the Con group, ^*∗*^*P* < 0.05, ^*∗∗*^*P* < 0.01 vs the CS control.

Th1/Th2 ratio keeps the dynamic balance and plays a critical role in regulating cell immunity and humoral immunity [33]. To examine whether HSPT regulated the CD4^+^ T cells subpopulation differentiation, we used flow cytometry to detect Th1 and Th2 subpopulation in spleen lymphocytes (Figures 11(a) and 11(b)). The proportion of CD4^+^ cells expressing IFN-*γ*, regarded as Th1 cells, notably increased in the CS group when compared to those mice in the Con group (Figure 11(c)). Additionally, the proportion of CD4^+^ T cells expressing IL-4, regarded as Th2 cells, also increased remarkably in the CS group (Figure 11(d)). As a consequence, the ratio of Th1/Th2 in the spleen (Figure 11(e)) was elevated to approximately 1.02-folds in the CS group when compared to the Con group. However, the administration of HSPT could decrease the proportion of CD4^+^IFN-*γ*^+^ and CD4^+^IL4^+^ T cells in a dose-dependent manner (Figures 11(a), 11(b)). The Th1/Th2 ratio (Figure 11(e)) was reduced by HSPT (M) and HSPT (H) to nearly Con levels. These results suggested that CS-induced imbalance of Th1/Th2 differentiation and HSPT could affect the inflammation by correcting the balance of Th1/Th2 subpopulations.

## 4. Discussion

There is a growing awareness that cigarette smoke exposure is associated with emphysema and chronic bronchitis in COPD patients [34, 35]. Chronic cigarette smoke exposure can result in inflammatory responses that may be an important precursor in the chronic changes to lung physiology [36]. Because animal models using cigarette smoke exposure are thought to be more similar to human COPD [37], our study used a mouse model of chronic cigarette smoke exposure to determine the impact of HSPT on modulating these alterations in the inflammatory response. Therefore, the present study aimed to investigate the outcome measurements that are impacted by chronic cigarette smoke exposure, including body inflammation resulting from smoke-induced bronchial injury, systemic inflammation, and immune cell activation.

Evidence shows that COPD results from chronic airway inflammation including a diversity of mast cells, involving EOSs, LYMs, NEUs, and MACs and epithelial cells [38–40]. The aforementioned cells release proinflammatory cytokine mediators that stimulate and promote airway inflammation, leading to predominantly small-airway disease, emphysema, and other chronic inflammatory diseases [41]. Consequently, interventions at an early stage with anti-inflammatory drugs that slow down the process of inflammatory changes may alleviate airway obstruction and inhibit the progression of airway remodeling. In the present study, the results demonstrated that HSPT intervention attenuated CS-induced inflammation in a mouse model of COPD. We found that HSPT inhibited the infiltration of inflammatory cells into the lung and reduced the influx of EOSs, LYMs, NEUs, and MACs in the BALF. These findings point to an important role for HSPT in preventing CS-induced inflammation.

CS-induced airway inflammation in COPD is associated with several inflammatory cytokines, such as IFN-*γ*, IL-2, IL-4, and IL-10 [42]. IFN-*γ* and IL-2, Th1-type cytokines, have been indicated to play an important role in the development of COPD following cigarette smoke exposure [43, 44]. IFN-*γ* and IL-2 secreted from Th1 cells activate LYMs and MACs to sites of inflammation [45]. IL-4 and IL-10, Th2-type cytokines, also have been identified as an important mediator of lung inflammation in COPD [46]. IL-4 can promote the EOSs aggregation in the sites of inflammation [47]. It is vitally important that blocking these responses prevents the pathological injury of CS exposure to mice [48–50]. Based on our previous findings, we propose researching the level of the above listed inflammatory cytokines (IFN-*γ*, IL-2, IL-4, and IL-10) in serum. We found that all these inflammatory cytokines are markedly elevated in the CS group which are in good agreement with those reported in the literature [47, 51, 52]. However, animals with the treatments of HSPT and NAC treatments significantly decreased the levels of the above listed inflammatory cytokines in the serum and lung. Moreover, the expression levels of IFN-*γ*, IL-2, IL-4, and IL-10 gene in the lung and spleen were also detected using qRT-PCR. We also found that these inflammatory cytokines mRNA were elevated in the CS group, and administration of HSPT or NAC obviously decreased the levels of the above listed inflammatory cytokines mRNA in the lung and spleen. There was a very good correlation between the splenic and pulmonic gene expression pattern and their actual concentrations in the serum and lung after pharmacological interventions.

Clinical studies have shown that the major cause of COPD is immune function disorder that increases the risk of inflammation [13, 14]. Immune function is the key to COPD treatment, and T lymphocytes can directly reflect the body's immune function status [53]. The spleen is the largest immune organ in the body and contains a large number of lymphocytes. Lymphocyte proliferation is an important index of the cellular immune response [54]. Based on a previous finding that HSPT could regulate the numbers of T cells to improve the body's immunity [25], we addressed whether HSPT mediates splenic lymphocyte proliferation induced by CS exposure. Flow cytometry was chosen to discuss the mechanism of HSPT on splenic lymphocyte proliferation. In our study, after 7 weeks of CS exposure, the proportion of CD3^+^CD4^+^ cells was elevated which was consistent with previous reports [55]. And Th1 and Th2 cells were also activated, as previously reported [14, 56]. However, after the administration of HSPT or NAC, the proportion of CD3^+^CD4^+^ T cells markedly decreased; in addition, the Th1 and Th2 cells decreased following a dose-dependent relationship. It is considered that T lymphocyte subsets and their cytokines are effectively controlled after pharmacological interventions. Further, we found that the imbalance of Th1/Th2 occurred in this study, and the Th1/Th2 ratio was shown to rise in the CS group. However, after the HSPT intervention, the Th1/Th2 ratio was declined to a near-normal level. In light of our findings here, HSPT revealed a potential immunomodulatory agent for the treatment of COPD.

## 5. Conclusion

The study demonstrated that HSPT exerted immunomodulatory effects on Th1/Th2 functionality in CS-induced COPD model mice. The findings indicate that HSPT may inhibit the development and severity of COPD, which have the potential to be further evaluated and developed as immunomodulatory agents for relative respiratory diseases.

## Figures and Tables

**Table 1:** The primers used for real-time quantitative PCR.

Name	Sequences
*β*-Actin	Sense: 5′-GGCTGTATTCCCCTCCATCG-3′
	Antisense: 5′-CCAGTTGGTAACAATGCCATGT-3′

IFN-*γ*	Sense: 5′-ATGAACGCTACACACTGCATC-3′
	Antisense: 5′-CCATCCTTTTGCCAGTTCCTC -3′

IL-2	Sense: 5′-TGAGCAGGATGGAGAATTACAGG-3′
	Antisense: 5′-GTCCAAGTTCATCTTCTAGGCAC-3′

IL-4	Sense: 5′-GGTCTCAACCCCCAGCTAGT -3′
	Antisense: 5′-GCCGATGATCTCTCTCAAGTGAT -3′

IL-10	Sense: 5′-GCTCTTACTGACTGGCATGAG-3′
	Antisense: 5′-CGCAGCTCTAGGAGCATGTG-3′

## Data Availability

The data used to support the findings of this study are included within the article.

## Abbreviations

COPD: Chronic obstructive pulmonary disease
HSPT: Hydrolyzed seawater pearl tablet
HPLC: Phenylisothiocyanate
PITC: High-performance liquid chromatography
CS: Cigarette smoking
NAC: N-acetylcysteine
BALF: Bronchoalveolar lavage fluid
H&E: Hematoxylin and eosin staining
EOSs: Eosinophils
LYM: Lymphocyte
MAC: Macrophage
NEU: Neutrophil
Th: Helper T
IFN: Interferon
IL: Interleukin
TNF: Tumor necrosis factor
qRT-PCR: Real-time quantitative reverse transcription-polymerase chain reaction
DEPC: Diethyl pyrocarbonate
FSC: Forward scattering
SSC: Side scattering.
